# Application of Modified Flanders Interaction Analysis During Mathematics Lessons in Lagos State Senior Secondary Schools

**DOI:** 10.12688/f1000research.166713.1

**Published:** 2025-10-01

**Authors:** Opesemowo Oluwaseyi Aina Gbolade, Taiwo Olufunmi, Alawaye Modupe, Etobro Benjamin Apkesi

**Affiliations:** 1 Mathematics, Science and Technology Education, University of Johannesburg Faculty of Education, Auckland Park, Gauteng, South Africa; 2 Educational Foundations and Counselling Psychology, Lagos State University Faculty of Education, Ojo, Lagos, Nigeria; 3 Educational Foundations and Counselling Psychology, Lagos State University Faculty of Education, Ojo, Lagos, Nigeria; 4 Educational Foundations and Counselling Psychology, Lagos State University Faculty of Education, Ojo, Lagos, Nigeria

**Keywords:** Classroom interaction, students’ participation, Flander interaction analysis categories system, modified Flanders interaction analysis, mathematics lessons.

## Abstract

**Objective:**

This study examined the application of Modified Flanders Interaction Analysis during mathematics lessons in senior secondary schools in the Festac area of Lagos State, Nigeria.

**Methods:**

The study employed a descriptive survey design to observe and analyse classroom interactions between teachers and students, focusing on verbal and non-verbal communication. Researchers used a structured observation schedule to collect data from a purposively selected sample of 10 mathematics teachers and 725 students across five schools. The researchers designed the instrument to collect information on teachers’ and students’ interaction patterns in the classroom. They analysed the data using mean scores, standard deviation, percentages, and t-test statistics, applying a 0.05 significance level for hypothesis testing.

**Findings:**

The results of the analysis revealed that teachers dominate all the activities in the classroom; that is, the teachers were the active people in the classes, while the students were just passive listeners and moderate engagement through non-verbal behaviours. Statistical analysis showed significant differences between teacher and student patterns, particularly verbal behaviours. The study underscores that mathematics classes in senior secondary schools in the Festac area of Lagos State were teachers-centered.

**Conclusion:**

Based on the study findings, the researchers recommended that mathematics teachers adopt more student-centered teaching approaches to enhance active student participation and engagement during lessons. Also, they should not be too strict, but they should be approachable, friendly, and accommodating so that the students will not be afraid to ask questions during or after the lesson, enhancing their performance. Hence, the government should ensure that teacher training programs incorporate observation techniques to effectively equip teachers with the skills to assess and improve classroom interaction.

## Introduction

The effectiveness of classroom instruction considerably depends on the quality of interaction between teachers and students. To foster meaningful classroom interaction in mathematics lessons is essential for enhancing deep learning and critical thinking. Engaging students through collaborative and active learning strategies not only aids in understanding abstract concepts but also promotes higher-order thinking skills ( Dominguez, 2024; Firdaus & Satriawan, 2025). The conventional teaching methods in many Nigerian secondary schools, including those in Lagos State, often rely on teacher-centered approaches that limit student participation and engagement. This raises concerns about students’ conceptual understanding and academic performance in mathematics ( Opesemowo, 2025a), the subject usually regarded as challenging and intimidating. Implementing more interactive teaching methods like group work, discussions, and hands-on activities could help mitigate these concerns and foster a more collaborative learning environment. By encouraging students to engage actively with the material and each other, teachers can promote better retention of concepts and greater problem-solving skills ( Mukhtoralieva, 2025; Xayrullayevna, 2024). This shift towards student-centered learning in mathematics classrooms in Lagos State can improve academic performance and cultivate a deeper appreciation and positive attitudes towards the subject among students.

Flander Interaction Analysis Categories System (FIACS), a structured framework for observing and analysing verbal communication in classrooms, was developed by Flanders (1970). This system categorises interactions into distinct types, allowing educators to effectively assess the dynamics of teacher-student communication ( Novianti & Anugrawati, 2023). It classifies classroom interactions into teacher talk, student talk, and silence. FIACS has been widely adopted in educational research for assessing instructional practices and promoting more balanced teacher-student interaction ( Ayunda et al., 2021; Nafisah & Setianingsih, 2024). However, the original FIACS was developed within a Western educational context ( Flanders, 1970), and its application in diverse settings such as Nigeria has unveiled certain limitations. This has led to the development and adoption of Modified Flanders Interaction Analysis (MFIA), which adapts the original framework to suit better local classroom realities, including larger class sizes, cultural norms, and varying levels of student preparedness.

The MFIA aims to categorise all the verbal actions that can be found in this study. The aim is to promote implementing the Flanders process in the educational process in schools, as the quantity and consistency of teacher-student interaction is a vital element of effective teaching and improved learning in the classroom. In Nigeria, several studies have shown that classroom interactions are heavily dominated by teacher talk, often exceeding 80% of total communication, leaving minimal space for students’ inquiry, discussion, and exploration ( Agbarakwe & Ona, 2024). This imbalance limits the development of critical thinking, problem-solving skills, and learner autonomy competencies increasingly emphasised in modern educational frameworks as required for 21st-century success. Mathematics, in particular, demands active student engagement, frequent feedback, and dialogue to clarify misconceptions and reinforce learning.

The application of MFIA offers a promising strategy to address these challenges by providing teachers with actionable insights into their communication patterns and enabling instructional adjustments that promote more student-centered practices. When properly implemented, MFIA can be a reflective tool for teachers to evaluate their interaction styles, increase student talk time, and foster a more inclusive and participatory learning environment. In the context of Lagos State, where senior secondary schools face varied educational challenges, including overcrowded classrooms and heterogeneous student backgrounds, applying MFIA during mathematics lessons may help bridge the gap between instructional intent and learning outcomes.

The teachers-students’ interaction was so poor that students could not freely relate with their teachers one-on-one. Another problem is the teacher-to-student ratio because of the large population of students. The higher the population, the more difficult it is for the teacher to have individual rapport with the students. Hence, most students lacked proper monitoring to understand the subject. According to Olayinka and Olayinka (2023), students’ attitudes toward mathematics significantly influence their performance, with negative attitudes often leading to poor outcomes. Mathematics is a fascinating subject, but some teachers know how to teach but do not know what to teach, while others know what to teach but do not know how to teach. These two sets of teachers will make mathematics difficult for students. A good mathematics teacher must know what to teach and how to teach. With this, his students will excel and develop a positive attitude towards the subject. However, some teachers today belong to the formal category and either know how or what to teach.

Basically, teachers play a pivotal role in shaping the students’ knowledge by understanding and managing the dynamic processes that influence student outcomes. Multiple factors determine these outcomes, including subject knowledge, instructional strategies, teaching experience, attitudes toward mathematics, professional development, and classroom climate. Recent studies highlight that effective teaching practices significantly impact students’ interest, self-efficacy, and achievement in mathematics ( Iwintolu et al., 2024; Zhu & Kaiser, 2022). Aligning instruction with students’ cognitive processes fosters a more profound understanding and promotes meaningful learning ( Kelly et al., 2023; Opesemowo, 2025). Moreover, student-centered pedagogical approaches that encourage active engagement, critical thinking, and problem-solving have also been shown to enhance academic performance. The successful implementation of such strategies depends on continuous professional development and institutional support ( Kong & Wang, 2024). Rather than simply transmitting knowledge, teachers act as facilitators of learning, guiding students through structured and interactive lessons. Tools like the MFIA help mathematics teachers reflect on classroom communication patterns and improve instructional practices, ultimately supporting better learning outcomes in mathematics.

Despite its potential, limited empirical studies have examined the use of MFIA in Nigerian secondary schools, especially within the subject area of mathematics. This study, therefore, seeks to explore the application of MFIA during mathematics lessons in senior secondary schools in Lagos State. Thus, an analysis of classroom interaction could provide a sensitive means of exposing how specific patterns in classroom verbal interaction reveal ways the teacher simulates and guides student learning. Hence, this study examines classroom interaction between teachers and students during mathematics lessons in senior secondary schools.

### Strategies for teaching Mathematics

The choice of teaching method is critical to instructional effectiveness and highly depends on the teacher’s understanding of individual student differences ( Felder & Brent, 2005). Understanding students’ diverse cognitive, emotional, and learning styles significantly enhances teachers’ ability to diversify instructional strategies. This insight allows teachers to tailor their teaching methods to better meet their students’ varied needs, ultimately advancing a more effective learning environment ( Babatimehin et al., 2025b; Opesemowo, 2024; Ramdani et al., 2022). Adopting varied teaching strategies can significantly enhance student engagement and learning outcomes in contemporary classrooms, especially in subjects like mathematics, where abstract reasoning is paramount ( Alam & Mohanty, 2024; Bray & Tangney, 2016; Rehman et al., 2024).

Teaching strategies often employed include the lecture method, which helps present large volumes of information; the laboratory method, which encourages hands-on learning and experimentation; and field trip methods, which help connect theoretical concepts to real-world experiences. Other commonly used approaches are the discussion methods, which foster interactive learning; the test method for assessment and feedback; and the problem-solving method, which promotes analytical thinking. Furthermore, the analytical, discovery, and expository methods cater to student independence levels and curriculum demands ( Magnusson & Zackariasson, 2019; Opesemowo et al., 2024). Mathematics teachers are therefore encouraged to adopt a flexible and reflective approach to teaching methods, aligning them with students’ needs, subject objectives, and classroom dynamics to promote effective and inclusive learning experiences.

### Statement of the problem

The persistent underachievement of students in mathematics within Nigerian secondary schools remains a significant concern. Despite various interventions, national examination results, such as those from the West African Examinations Council (WAEC) and National Examinations Council (NECO), consistently indicate low proficiency levels among mathematics students ( Akinpelu et al., 2024; Babatimehin et al., 2025a). This trend is particularly evident in Lagos State, where students’ performance in mathematics continues to lag behind expectations. A critical examination of teaching methodologies reveals a predominant reliance on teacher-centered approaches, characterised by rote learning and minimal student engagement. Such methods often neglect the diverse learning needs of students and fail to foster critical thinking skills essential for mathematical problem-solving ( Awofala, 2017). Moreover, traditional assessment techniques, primarily through questionnaires and test scores, provide limited insight into the actual classroom dynamics and the quality of teacher-student interactions. ( Akinpelu et al., 2024).

In addition, observational studies employing tools like FIACS have highlighted the dominance of teacher talks over student participation in classrooms. For instance, a study conducted at the Federal College of Education (Technical), Omoku, Rivers State, Nigeria, found that teacher talk accounted for approximately 83.43% of classroom interaction, and student talk was a mere 12.71% ( Agbarakwe & Ona, 2024). Such imbalances in classroom interactions can hinder the development of a conducive learning environment and impede students’ mathematical understanding. Given these challenges, a pressing need exists to explore and implement more interactive, student-centered teaching strategies in mathematics lessons. The application of MFIA during mathematics lessons offers a promising avenue to assess and enhance classroom interactions, potentially improving student engagement and performance in mathematics.

### Purpose of the study

To assess the classroom interaction pattern of teaching and learning mathematics in Amuwo-Odofin Local Government of Lagos. Specifically, the study examined whether mathematics class was student-centered or teacher-centered and the adequacy of classroom interaction between the teachers and the students.

### Research questions

1. What percentage of the time is spent on each classroom activity?
2. What percentage of the time is spent on each classroom behaviour?

### Research hypotheses

1. There is no significant difference between the verbal behaviour of teachers and students.
2. There is no significant difference between the non-verbal behaviour of teachers and students.
3. There is no significant difference between the time of interaction of teachers and students.

## Methodology

This study adopted a descriptive survey research design, which is appropriate for systematically observing and describing the interaction patterns between teachers and students during classroom instruction ( Creswell & Creswell, 2017). This approach allows researchers to capture the nuances of verbal exchanges and the dynamics of classroom interactions, providing insight into how this pattern influences learning outcomes.

### Population

The target population was comprised of mathematics teachers and students in the Festac area, Lagos State, Nigeria. The study sample consisted of 10 mathematics teachers and 725 students from five public secondary schools in the Festac area of Lagos State, Nigeria. We utilised a purposive sampling technique to select the participants, targeting schools with an existing record of consistent mathematics instruction and an adequate student population in the state.

### Instrument

The study instrument employed was a structured classroom observational schedule adopted from the MFIA, which allows for categorising and analysing verbal and non-verbal communication in teaching-learning environments. The observational instrument, the classroom activity sheet, was used to gather data on classroom interactions at one-minute intervals during live mathematics lessons. The instrument was structured into 10 categories in line with the FIACS and further grouped into four segments: teacher verbal behaviour, student verbal behaviour, teacher non-verbal behaviour, and student non-verbal behaviour. This structure allowed for a comprehensive assessment of the quantity and quality of interaction in the mathematics lesson.

### Data collection

The study analysed the collected data quantitatively using descriptive statistics such as mean scores, standard deviation, and percentages to identify trends in teacher-student interaction. Additionally, inferential statistics, specifically the t-test, were used to determine the significance of observed differences at the 0.05 significance level. These statistical methods ensure a robust interpretation of findings and allow the researcher to conclude the effectiveness of classroom interaction patterns on students’ learning experiences in mathematics.

## Results

Question 1: What percentage of the time is spent on each classroom activity?

The results for the analysis of the percentage of the time spent are given in Table 1.

**Table 1.** Time spent on each classroom activity.

S/N	Class activities	Time spent in minutes	Percentage
1	Praise and encouragement	1	2.50
2	Asking questions about content	1.50	3.75
3	Teaching	14.50	30.50
4	Give direction	0.80	2.00
5	Student responds to teacher	4.40	11.00
6	The teacher responds to the student	0.80	2.00
7	Teacher non-verbal behaviour (writing and drawing)	6.60	16.50
8	Students non-verbal behaviour	6.10	15.25
9	Silence in the class	0.60	1.50
10.	Confusion - noise in the class	3.60	9.00

Table 1 analyses the distribution of instructional time across various classroom activities during mathematics lessons. The highest proportion of time (14.50), 30.50%, was devoted to direct teaching, indicating a teacher-centered approach dominated the classroom interaction, which aligns with conventional instructional practices in mathematics lessons. This was followed by teacher non-verbal behaviour, such as writing and drawing on the board, which accounted for 16.5% of classroom time, and student non-verbal behaviour, which constituted 15.25%. These findings suggest that a significant portion of the lesson was devoted to individual cognitive engagement, albeit largely passive.

The student responses to teacher questions represented 11.0% of the observed time, indicating moderate verbal student participation. Conversely, teacher responses to students and giving directions were relatively low, each accounting for only 2.0%, implying limited dialogic interaction or feedback within the lesson. Other forms of teacher talk, such as praise and encouragement (2.5%) and content-related questioning (3.75%), were used sparingly, potentially reducing opportunities for formative assessment and student motivation. The classroom also experienced 3.6 minutes of confusion or noise and 0.6 minutes of silence, suggesting occasional disruptions that may affect instructional efficiency. Finally, the time allocation reflects a predominantly teacher-based classroom with limited interactive and feedback-driven engagement, highlighting the need for more student-centered approaches that could promote active student engagement, increased dialogue and diversified instructional strategies to support meaningful learning.

Question 2: What percentage of the time is spent on each classroom behaviour?

Data was analysed using the classroom activities tables, which were regrouped into four: teacher verbal behaviour, teachers’ non-verbal behaviour, students’ verbal behaviour and students’ non-verbal behaviour. The time slice allotted to each classroom is shown in Table 2.

**Table 2.** Time spent on classroom behaviour.

S/N	Classroom behaviour	Time spent in minute	Percentage
1	Teachers’ verbal	17.90	44.75
2	Students’ verbal	8.80	22.00
3	Teachers’ non-verbal	6.60	16.5
4	Students’ non-verbal	6.70	16.75

Table 2 shows time spent on classroom behaviour during mathematics lessons. The data indicates that teachers’ verbal behaviour dominates, occupying 44.75% of classroom time, reflecting a teacher-centered instructional style. Students’ verbal behaviour accounts for 22%, suggesting moderate student participation but limited dialogic interaction. Non-verbal behaviours are almost equally distributed, with teachers’ non-verbal behaviour at 16.5% and students’ non-verbal behaviour at 16.75%, indicating shared time spent on tasks such as writing or working independently.

Hypothesis 1: There is no significant difference between the verbal behaviour of teachers and students.

Table 3 presents a t-test comparison of the verbal behaviour of teachers and students during mathematics lessons. The result shows that teachers had a higher mean verbal behaviour score (M = 17.90, SD = 3.03) than students (M = 5.20, SD = 1.62). The calculated t-value (t = 11.087) exceeds the critical t-value (t = 2.262) at df = 9 and p < 0.05, indicating a statistically significant difference. Therefore, we rejected the null hypothesis. This suggests that teachers dominate classroom verbal interactions significantly more than students, reinforcing a teacher-centered communication pattern.

Hypothesis 2: There is no significant difference between the non-verbal behaviour of teachers and students.

**Table 3.** To test this hypothesis, a t-test comparison of the verbal behaviour of teachers and students.

Variable	N	X	SD	df	t.cal.	t.table	remark
Verbal behaviour of teachers	10	17.90	3.027	9	11.087	2.262	Rejected
Verbal behaviour of students	10	5.20	1.619				

* P < 0.05.

Table 4 shows the independent t-test of the non-verbal behaviour of teachers and students during classroom instruction. The mean score (M = 6.60, SD = 1.647) for teachers’ non-verbal behaviour is higher than the student’s (M = 6.10, SD = 2.283). The calculated t-value (t = 0.745) is less than the critical t-value (t = 2.262) at df = 9 and 0.05 significant level, indicating that the difference is not statistically significant. Therefore, the null hypothesis is accepted, suggesting that teachers and students engage in non-verbal classroom behaviours at comparable levels.

**Table 4.** To test this hypothesis, a t-test comparison of the non-verbal behaviour of teachers and students.

Variable	N	X	SD	df	t.cal.	t.table	Remark
Non-verbal behaviour of teachers	10	6.60	1.647	9	0.745	2.262	Accepted
Non-verbal behaviour of students	10	6.10	2.283				

* P < 0.05.

Table 5 presents a t-test comparison of the interaction time between teachers and students during classroom instruction. The mean interaction time for teachers is 24.50 minutes, while that of students is 11.30 minutes. The calculated t-value (t = 8.337) exceeds the critical value (t = 2.262) at df = 9 and p < 0.05 significant level, indicating a statistically significant difference. Hence, we rejected the null hypothesis. This result suggests that teachers dominate classroom interaction time, highlighting a teacher-centered instructional pattern with limited student participation.

**Table 5.** T-test table of interaction time between teachers and students.

Variable	N	X	SD	df	t.cal	t.table	Remark
Interaction time of teachers	10	24.50	4.301	9	8.337	2.262	Rejected
Interaction time of students	10	11.30	3.199				

* P < 0.05.

## Discussion

This study is based on the application of MFIA, offering valuable insights into classroom interaction patterns during mathematics instruction in Lagos State senior secondary schools. The results collectively point to a predominantly teacher-centered approach, with limited student verbal engagement and interaction.

This study observed that teachers spend much of class time engaging in verbal instruction. This aligns with the findings of Burgess et al. (2020), who emphasised that heavy reliance on teacher talk can diminish opportunities for student engagement, critical thinking, and mathematical discourse. While helpful in delivering content, such dominance may hinder deeper understanding, especially if not complemented by student-centered learning activities. Similarly, Blatchford et al. (2011) noted that when teachers dominate classroom talk, students are often relegated to passive roles, limiting their active capacity, participation and intellectual autonomy. Subsequently, students’ verbal contributions were relatively minimal compared to their teachers. This finding is consistent with Chen et al. (2020), who argued that restricted student talk time can result in lower cognitive engagement and hinder the development of mathematical reasoning. Moreover, low verbal participation may reflect classroom culture where students are not encouraged or supported to voice their ideas, a concern also highlighted by Ho et al. (2023) in their study on silence over the wire: student verbal participation and the virtual classroom in the digital era. While some cultural norms may discourage verbal participation, Karjanto (2019) stresses the importance of gradually scaffolding students toward more active classroom involvement, even in traditionally teacher-centered contexts.

Teachers and students exhibited relatively balanced non-verbal behaviour, including writing, drawing, and not-taking. This parity reflects mutual involvement in academic tasks, suggesting that non-verbal engagement is more equitably distributed while verbal interaction may be one-sided. Ijaz et al. (2023) have argued that teachers’ effective use of non-verbal cues like gestures and visual aids can significantly enhance student comprehension. However, Valenzeno et al. (2003) caution that ambiguous or mismatched non-verbal gestures can confuse students, stressing the need for purposeful use of such cues. Therefore, teachers must be mindful of their non-verbal communication in the classroom, as it plays a key role in facilitating student learning and understanding. By being intentional and transparent with their gestures and visual aids, teachers can create a more engaging and effective learning environment for their students. Ultimately, the combination of verbal and non-verbal communication in the classroom can lead to improved academic performance and student success.

The disparity in the overall interaction time between teachers and students further confirms the teacher-dominated nature of classroom discussions. Research by Lo and Chen (2021) suggests that students benefit more from learning environments where their voices are integral to the instructional process. When the teacher-centered approach monopolises classroom time, opportunities for students to develop problem-solving skills, articulate reasoning, and collaborate with peers are constrained. To create a more student-centered learning environment, teachers should actively seek input from their students and provide opportunities for them to engage in discussions and activities that promote critical thinking and collaboration. By allowing students to take more ownership of their learning experiences, teachers can help facilitate the development of essential skills that are crucial for success in both academic and real-world settings. Encouraging active participation and valuing the perspectives of each student can lead to a more inclusive and dynamic educational experience for everyone involved.

## Conclusion

The result of the study showed that teaching mathematics in senior secondary school has not completely weaned itself from the historical antecedent in which teachers dominated classroom activities. Again, the study highlights a significant imbalance in teacher-student interactions during mathematics lessons, with teachers occupying the dominant communicative role. While non-verbal communication engagement is more balanced, the lack of substantial student verbal participation raises concerns about the effectiveness of current teaching practices in fostering deep understanding and critical thinking. To address these issues, teachers should adopt dialogic teaching strategies, leverage technological tools to promote participation and engage in professional development focused on enhancing classroom discourse. Such measures are essential for creating learning environments that support active student engagement and improved learning outcomes in mathematics lessons.

### Recommendations

Based on the findings of this study, we recommended that:

• Mathematics teachers should not be too strict and must be friendly, approachable, and accommodating so that the students will not be afraid to ask questions during and after the lesson.
• Classroom observational techniques have gained worldwide recognition in developed and developing countries, and the government should make efforts to include observational techniques in teacher training institutions.
• In-service teachers’ seminars should be organised for such teachers to expose them to the implications of classroom interaction.
• Workshops, seminars, training, and conferences should be organised regularly to enhance professional development.

### Limitations and suggestions for further study

This study was limited to a small number of secondary schools within the Festac area of Lagos State, Nigeria, which restricts the generalizability of the findings to other educational contexts. The purposive sampling method and reliance on observational data, time constraint and reliance on single data may have introduced bias and failed to capture deeper cognitive or affective aspects of classroom interaction. In addition, the study focused solely on mathematics lessons, excluding other subject areas that might offer comparative insights. For further research, broader samples across diverse regions and subjects are recommended. Incorporating mixed methods, such as interviews and student feedback, could provide richer insights into the dynamics of classroom interaction.

### Ethical statement

Ethical approval for this study was obtained from the Faculty of Education Ethics Committee at Lagos State University, Ojo, Nigeria. The study ethical number is Ethical Clearance Number: S E M 3-2 0 2 5-1 2 3 4. The study was conducted in compliance with the principles outlined in the Declaration of Helsinki. All participants were fully informed about the purpose, scope, and procedures of the research before data collection commenced. Informed consent was obtained from each participant, ensuring their voluntary participation. To safeguard confidentiality, all responses were anonymized, and personal identifiers were removed. The data were treated with strict confidentiality, and measures were taken to ensure that participants’ privacy, dignity, and trust were maintained throughout the study.

### Informed consent

As part of the research process, we obtained written informed consent from all participants prior to data collection. The participants in this study were senior secondary students aged 18 years and above and therefore were not minors. Each participant was fully informed about the purpose of the study, the voluntary nature of their participation, and their right to withdraw at any time without penalty. They were also assured that the information provided would be used strictly for research purposes, that their identities would remain confidential, and that all data would be anonymised during analysis and reporting. Since no minors were involved in the study, parental or guardian consent and child assent were not applicable. By ensuring full transparency and respecting participants’ rights, the research process upheld the principles of ethical integrity and maintained participants’ trust, thereby strengthening the overall credibility of the study.

### Clinical trial number

Not applicable.

## Data Availability

MFIA_Date set https://doi.org/10.5281/zenodo.15698071 ( Opesemowo, Oluwaseyi Aina Gbolade, 2025b) MFIA Questionnaire https://doi.org/10.5281/zenodo.17049546 ( Opesemowo, Oluwaseyi Aina Gbolade, 2025c) Data are available under the terms of the Creative Commons Attribution 4.0 International license (CC-BY 4.0).
