# Supplementary material for: Application of Modified Flanders Interaction Analysis During Mathematics Lessons in Lagos State Senior Secondary Schools
Source: F1000Res. 2025 Dec 2;14:1018. Originally published 2025 Oct 1. [Version 2] doi: 10.12688/f1000research.166713.2 (PMC12790597; doi:10.12688/f1000research.166713.2)
Supplement: Peer review history

# Reviewer report: Reviewer response for version 2

**DOI:** 10.5256/f1000research.192113.r437946

**Published:** 2026-07-22

**Authors:** Torang Siregar

**Affiliations:** 1 UIN Syekh Ali Hasan Ahmad Addary Padangsidimpuan, Sumatra, Indonesia

**Reviewer Report**

 Thank you for revising the manuscript. The authors have adequately addressed the reviewers' comments, and the manuscript is now clearer and better organized. I confirm my original decision of **Approved** and recommend the manuscript for indexing.

Is the work clearly and accurately presented and does it cite the current literature?

Yes

If applicable, is the statistical analysis and its interpretation appropriate?

Yes

Are all the source data underlying the results available to ensure full reproducibility?

Yes

Is the study design appropriate and is the work technically sound?

Partly

Are the conclusions drawn adequately supported by the results?

Yes

Are sufficient details of methods and analysis provided to allow replication by others?

Partly

Reviewer Expertise:

Mathematics Education, STEM Education, RME, AI in Education, Educational Technology Integration, Discovery Learning Models, Critical Thinking in Mathematics, Meta-Analysis in Education, Computer-Assisted Mathematics Learning, Mathematics Education, Educational Technology Integration, Discovery Learning Models, Critical Thinking in Mathematics, Meta-Analysis in Education, Computer-Assisted Mathematics Learning

I confirm that I have read this submission and believe that I have an appropriate level of expertise to confirm that it is of an acceptable scientific standard.

# Reviewer report: Reviewer response for version 2

**DOI:** 10.5256/f1000research.192113.r439353

**Published:** 2026-01-10

**Authors:** Fidele Ukobizaba

**Affiliations:** 1 African Centre of Excellence for Innovative Teaching and Learning Mathematics and Science (ACEITLMS), University of Rwanda College of Education, Kayonza, Rwanda

The revised manuscript shows clear improvement across several sections.  The presentation of results is now more organized and easier to follow.

 However, some points need to be addressed before acceptance for indexing.

- First, the results in Table 2 are under objective 2. Yet, Table 2 came before. Thus, Table 2 should come after objective 2.
- Second, the remark in Table 4 was to accept null hypothesis (H0). Yet, it was shown that the p-value is less than 0.05. Which means that the H0 is to be rejected.
- Third, the computed p-values for Table 3,4, and 5 should be presented in tables.
- Fourth, the conclusion made (first recommendation) should be removed since there are not findings supporting it.

Once the above points have been adequately addressed, the article will be improved for coherence, readability, and academic rigor.

Is the work clearly and accurately presented and does it cite the current literature?

Yes

If applicable, is the statistical analysis and its interpretation appropriate?

Partly

Are all the source data underlying the results available to ensure full reproducibility?

Yes

Is the study design appropriate and is the work technically sound?

Partly

Are the conclusions drawn adequately supported by the results?

Partly

Are sufficient details of methods and analysis provided to allow replication by others?

Yes

Reviewer Expertise:

Mathematics Education, applied calculus, hands on activities in mathematics, project-based learning, mathematics pedagogy, and educational research methods.

I confirm that I have read this submission and believe that I have an appropriate level of expertise to confirm that it is of an acceptable scientific standard, however I have significant reservations, as outlined above.

# Reviewer report: Reviewer response for version 2

**DOI:** 10.5256/f1000research.192113.r443413

**Published:** 2026-01-09

**Authors:** Taha Kuzu

**Affiliations:** 1 University of Education Schwäbisch Gmünd, Schwäbisch Gmünd, Germany

Review for the article with the title

*Application of Modified Flanders Interaction Analysis During Mathematics*

*Lessons in Lagos State Senior Secondary Schools*

 Dear authors,

 thank you for the opportunity to review your article *„Application of Modified Flanders Interaction Analysis During Mathematics Lessons in Lagos State Senior Secondary Schools“.* In my opinion, the manuscript addresses a relevant and empirically important issue, namely classroom interaction patterns in senior secondary mathematics classrooms in Lagos State, and it is commendable that you seek to adapt an established observational instrument to a specific local context. In the following, I focus my review on the introduction and research gap, the theoretical and conceptual framing (especially regarding FIACS/MFIA), the method, and the empirical insights, with particular attention to points where the argumentation could be sharpened or deepened.

    With regard to **the introduction and research gap**, you convincingly motivate the general relevance of classroom interaction for learning processes in mathematics and situate your study within concerns about teacher-centred instruction in Nigerian secondary schools. For example, you argue that *“the conventional teaching methods in many Nigerian secondary schools… often rely on teacher-centered approaches that limit student participation and engagement”*. At this point, however, a very critical reader might perceive a risk of confirmatory bias: teacher-centredness is introduced early on as a defining characteristic of the context and as a pedagogical problem, while the empirical analysis that follows primarily serves to document and reaffirm this assumption rather than to interrogate it in a more differentiated or open-ended manner. I would therefore suggest omitting or at least substantially weakening this assumption at the introductory stage, as it is not yet empirically substantiated at the point at which it is introduced. Leaving the question of teacher-centredness more open in the introduction would allow the empirical analysis to function more clearly as an exploratory investigation rather than as a confirmation of a prior claim.

    Another issue is that the introduction remains somewhat broad and normative in tone, and the specific research gap could be articulated more sharply. While you state that *“limited empirical studies have examined the use of MFIA in Nigerian secondary schools, especially within the subject area of mathematics”*, it would strengthen the paper to specify more clearly what is not yet known: Is it the mere distribution of talk, the balance between verbal and non-verbal behaviour, or the didactical quality of interaction patterns in mathematics classrooms? At present, the reader is led quite early toward the expectation that teacher-centredness (whatever that means, see below) is problematic per se (and expectable, see above), but the analytical added value of documenting this again remains somewhat implicit. What would clearly strengthen the article is a (more) explicit definition of *“teacher-centredness”*. It would be important to clarify under which conditions an interaction pattern or instructional process is classified as teacher-centred and, equally, when this label may not be appropriate. In particular, a high proportion of teacher turns does not necessarily indicate teacher-centred instruction in a didactically problematic sense. For example, a high proportion of teacher talk does not necessarily indicate transmissive or authoritarian instructional practices; it may instead reflect supportive or process-oriented functions such as “Giving Directions” (FIACS Category 4). From a didactical perspective, this form of interaction – even if it occurs in a high amount – can constitute a meaningful and pedagogically justified form of instructional support, rather than an indicator of reduced learning quality (because of being too „teacher-centered“). Without such conceptual clarification, there is a risk that teacher-centredness is equated too directly with the sheer quantity of teacher talk, rather than being interpreted in relation to the function and quality of the respective interactional moves.

    This leads directly to the **theoretical background and conceptual framing**, particularly concerning FIACS and its modification. You correctly outline FIACS as a framework focusing on verbal classroom interaction and acknowledge that it was developed in a Western context. You also state that MFIA was “modified to better reflect the realities of Nigerian mathematics classrooms, where teacher-dominated instruction, board work, and limited student-initiated talk are common” (p. 5). From a conceptual perspective, this sentence is problematic in two ways. First, it risks circularity: teacher-dominated instruction is described as a “reality” that motivates the modification of the instrument, and the study then empirically shows that instruction is teacher-dominated. Second, the notion of “modification” itself remains underspecified on a theoretical level. While you later list and clearly define the ten categories used in MFIA, these categories largely correspond to the original FIACS categories, albeit with refined labels and the explicit inclusion of non-verbal behaviour. A more explicit discussion of what is theoretically gained by calling this an expanded or modified version would be helpful. Is the modification primarily empirical-pragmatic, or does it also imply a different understanding of classroom interaction? At present, the (critical) reader may ask: What exactly is modified beyond contextual relabelling and the inclusion of non-verbal behaviour?

    Relatedly, while you rightly mention common critiques of FIACS indirectly by noting its focus on interaction quantity, the manuscript could benefit from a more explicit engagement with these limitations. FIACS (and MFIA) primarily capture what happens in terms of interaction *distribution*, not why it happens or how cognitively demanding or didactically meaningful teacher moves are. This limitation becomes particularly relevant in light of your conclusions (see below).

    Turning to the **method**, the revised version has clearly improved in transparency and rigour. The detailed description of observer training, calibration, and inter-rater reliability (with κ = 0.81) is a clear strength of the manuscript. The observation protocol, lesson duration, and number of observations per teacher are also clearly specified. One point that remains conceptually underdeveloped, however, concerns the analytical scope of the method. Given that MFIA codes interaction at one-minute intervals and aggregates frequencies and percentages, the design is inherently descriptive. This is not a weakness per se, but it should be acknowledged more explicitly as a methodological boundary. At several points, the text implicitly suggests that interaction patterns can be linked to (deeper) learning processes (e. g., when stating that *„applying MFIA during mathematics lessons may help bridge the gap between instructional intent and learning outcomes“*), yet the method does not allow access to cognitive activation, quality of feedback, or students’ meaning-making processes.

    This becomes most evident in the **empirical results and their interpretation**. Your key finding – that teachers dominate verbal classroom interaction while students are largely passive listeners with moderate non-verbal engagement – is clearly presented and statistically well supported. For instance, you show that teachers’ verbal behaviour accounts for 44.75% of classroom time compared to 22% for students. However, the central interpretive question remains: What do we actually gain from knowing that instruction is labelled as *“teacher-centred”* on this basis? As a reader, I miss a deeper engagement with the *quality* and function of teacher talk. Your own Table 1 differentiates between various types of teacher interaction, such as “Direct Teaching/Lecturing” (30.50%) and “Giving Directions” (2.00%) on the one hand, and more dialogic categories like “Content-Related Questioning” (3.75%) and “Teacher Response to Students” (2.00%) on the other. These distributions are highly informative, yet they are not analysed in depth or interpreted didactically. In addition, one aspect that may confuse readers concerns the category *“Criticism and Authority Cues”*. While this category is introduced as part of the MFIA framework on page 6, it does not appear explicitly in Table 1. This raises the question of whether this category was empirically absent or whether it was subsumed under another category. If the former is the case, the implication would be that no critical or authority-based teacher behaviour occurred at all, which seems implausible and therefore requires clarification.

    From a didactical perspective, it makes a crucial difference whether teacher dominance consists primarily of high-quality explanations, cognitively activating questions, or scaffolding feedback, or whether it is mainly organisational, directive, or authority-based talk. As you note in passing, categories such as “Giving Directions” and “Teacher Response to Students” occur relatively rarely, but the implications of this are not fully unpacked. Without such an analysis, the conclusion that teaching is teacher-centred risks remaining descriptively correct (if only the speaking percentages/ turn-taking amount is considered) but educationally underspecified. Teacher-centredness can mean very different things in mathematics education: it may involve carefully designed impulses aimed at cognitive conflict and conceptual change, or it may consist of procedural instruction focused on right and wrong answers without engaging with students’ learning processes.

    This issue also affects your recommendations and discussion. You conclude that teachers should adopt “more student-centered teaching approaches to enhance active student participation”. While this aligns with curriculum ideals and international standards, it is important to clarify that more student talk or participation does not automatically imply higher cognitive activation. Increasing the quantity of student turns without attention to task quality, mathematical substance, or feedback processes may result in superficial participation. Your own data could be used more productively by discussing which MFIA categories should be strengthened from a didactical standpoint and for what reasons. For example, interactional moves classified as *“Giving Directions”* could be deliberately expanded if the aim is to establish a more process-oriented and supportive feedback culture in the classroom, rather than merely increasing the overall amount of student or teacher talk.

    Finally, regarding the **limitations**, it is positive that you explicitly acknowledge that the study *“may have introduced bias and failed to capture deeper cognitive or affective aspects of classroom interaction”* and suggest mixed-methods approaches for future research. I would encourage you to be even more explicit here: cognitive aspects are not merely imperfectly captured by MFIA; they are largely outside the analytical scope of the instrument. Stating this more clearly would not weaken the study but rather position it more honestly as a descriptive, observation-based contribution that can inform further, more process-oriented research.

 In sum, the study is methodologically careful and empirically solid in documenting interaction patterns, but its analytical and didactical contribution would be significantly strengthened by (a) a clearer conceptualisation of what is modified in MFIA, (b) a deeper analysis of the quality of teacher talk using your own category system, and (c) a more nuanced discussion of what “teacher-centred” and “student-centred” mean from a learning-theoretical perspective. Addressing these points would greatly enhance the explanatory power and educational relevance of the manuscript.

Is the work clearly and accurately presented and does it cite the current literature?

Yes

If applicable, is the statistical analysis and its interpretation appropriate?

Partly

Are all the source data underlying the results available to ensure full reproducibility?

Yes

Is the study design appropriate and is the work technically sound?

Yes

Are the conclusions drawn adequately supported by the results?

Partly

Are sufficient details of methods and analysis provided to allow replication by others?

Partly

Reviewer Expertise:

Mathematics Education, Multilingualism/ Translanguaging, Interaction Analysis, Primary Education, Digital Media in Education

I confirm that I have read this submission and believe that I have an appropriate level of expertise to confirm that it is of an acceptable scientific standard, however I have significant reservations, as outlined above.

# Reviewer report: Reviewer response for version 1

**DOI:** 10.5256/f1000research.183745.r432297

**Published:** 2025-11-22

**Authors:** Torang Siregar

**Affiliations:** 1 UIN Syekh Ali Hasan Ahmad Addary Padangsidimpuan, Sumatra, Indonesia

**Final Assessment and Required Revisions**

 Overall, this is a valuable contribution to educational research in Nigeria, highlighting persistent challenges in classroom interaction during mathematics instruction. The study addresses an important gap and uses a recognized analytical framework.

**Points That Must Be Addressed Before Acceptance**:

1. Clarify the MFIA Categories Used: Provide a list of the 10 behavioral categories adapted in the study, explaining any modifications from the original FIACS.
2. Detail Observer Training and Reliability: Describe how observers were trained and whether inter-rater reliability was measured (e.g., percentage agreement or Cohen’s kappa).
3. Specify Observation Protocol: State how many lessons were observed per teacher, total observation duration, and scheduling (e.g., single or multiple days).
4. Improve Citation Formatting: Ensure all references follow a consistent style (APA) and remove duplicates or formatting errors.
5. Link Instrument to Text: Mention the questionnaire DOI in the Methods section, not just in data availability.

Once these issues are resolved, the article will be scientifically robust and suitable for indexing.

***Recommendation***: **Accept after Minor Revisions**

Is the work clearly and accurately presented and does it cite the current literature?

Yes

If applicable, is the statistical analysis and its interpretation appropriate?

Yes

Are all the source data underlying the results available to ensure full reproducibility?

Yes

Is the study design appropriate and is the work technically sound?

Partly

Are the conclusions drawn adequately supported by the results?

Yes

Are sufficient details of methods and analysis provided to allow replication by others?

Partly

Reviewer Expertise:

Mathematics Education, Educational Technology Integration, Discovery Learning Models, Critical Thinking in Mathematics, Meta-Analysis in Education, Computer-Assisted Mathematics Learning, Mathematics Education, Educational Technology Integration, Discovery Learning Models, Critical Thinking in Mathematics, Meta-Analysis in Education, Computer-Assisted Mathematics Learning

I confirm that I have read this submission and believe that I have an appropriate level of expertise to confirm that it is of an acceptable scientific standard.

# Response

**Published:** 2025-11-23

**Authors:** Oluwaseyi Opesemowo

**Affiliations:** Mathematics, Sci, & Tech. Edu., University of Johannesburg Faculty of Education, Auckland Park, Gauteng, South Africa

**Instrument**

 The study employed the MFIA instrument (https://doi.org/10.5281/zenodo.17049546) to systematically capture teacher–student interaction patterns during mathematics instruction. This allows for categorising and analysing verbal and non-verbal communication in teaching-learning environments. The observational instrument, the classroom activity sheet, was used to gather data on classroom interactions at one-minute intervals during live mathematics lessons. The MFIA was adapted from the classic FIACS but modified to better reflect the realities of Nigerian mathematics classrooms, where teacher-dominated instruction, board work, and limited student-initiated talk are common. The MFIA used in this study retained the ten original FIACS behavioural categories but introduced contextual modifications. The categories are: (1) Praise and Encouragement – Teacher statements or gestures showing approval, motivation, or positive reinforcement. (2) Content-Related Questioning – Teacher questions directly related to mathematics concepts, procedures, or problem solving. (3) Direct Teaching/Lecturing – Explanations, demonstrations, worked examples, definitions, and conceptual exposition. (4) Giving Directions – Instructions relating to tasks, class activities, behaviour, or note-taking. (5) Teacher Response to Students – Clarifying, expanding, or giving feedback on student responses. (6) Criticism and Authority Cues – Statements reflecting correction of behaviour or implicit assertion of authority. (7) Teacher Non-Verbal Behaviour – Writing on the board, gesturing, using teaching aids, or other physical demonstrations. (8) Students non-verbal behaviour – Students answering teachers’ questions or giving short non-verbal responses. (9) Student-Initiated Talk – Questions, explanations, or ideas introduced spontaneously by students. (10) Confusion and Noise – Pauses, classroom noise, disruptions, or uncertainty during instruction.

**Observer Training and Reliability**

 To ensure accuracy, consistency, and credibility in applying the Modified Flanders Interaction Analysis (MFIA) instrument during classroom observations, two observers underwent a structured training programme prior to data collection. The training process was conducted over two weeks and consisted of three sequential phases designed to familiarise observers with the MFIA categories and strengthen their coding precision.

 Phase 1: Familiarisation with MFIA Categories

 Observers were introduced to the ten behavioural categories adapted for the study, with emphasis on distinguishing verbal and non-verbal behaviours. They reviewed sample video lessons, practised identifying teacher and student behaviours, and discussed coding boundaries to ensure shared understanding of all category definitions.

 Phase 2: Guided Practice and Calibration Exercises

 Observers independently coded the same set of practice lessons at one-minute intervals and later compared results. Discrepancies were discussed and resolved collaboratively, enabling the observers to refine their decision rules and achieve a uniform interpretation of the MFIA categories. Calibration continued until agreement was consistently above the acceptable threshold for observational studies.

 Phase 3: Certification and Field Simulation

 Before proceeding to live classroom observations, each observer completed a certification exercise by coding two additional mathematics lesson videos. These were compared against a master coding guide prepared by an experienced MFIA user. Only after demonstrating acceptable accuracy were they approved for field data collection.

**Inter-Rater Reliability**

 To quantify agreement between observers during the pilot stage, percentage agreement and Cohen’s kappa (κ) were computed. Across all MFIA categories, observers achieved:

 • Percentage Agreement: 87%

 • Cohen’s Kappa (κ): 0.81

 A kappa coefficient above 0.80 indicates strong inter-rater reliability, confirming that the observers consistently applied the MFIA coding scheme. Any minor inconsistencies identified were resolved prior to the commencement of formal data collection. This process ensured that the observational data collected during classroom visits were both reliable and valid for analysis.

**Observation Protocol**

 Each teacher was observed teaching two separate mathematics lessons, allowing for comparison of interaction patterns across different content areas and classroom conditions. Each lesson observation lasted 40 minutes, consistent with the standard lesson duration in the participating schools. Observations were scheduled across two different days for each teacher to minimise the influence of one-off events and to capture more stable behavioural patterns. The observation schedule was arranged in collaboration with school administrators to ensure that lessons selected reflected typical classroom practice rather than special or pre planned activities. This approach provided a total observation time of 80 minutes per teacher, generating sufficient data for reliable MFIA coding and analysis.
